# Repair of monolithic zirconia restorations with different direct resin composites: effect on the fatigue bonding and mechanical performance

**DOI:** 10.1007/s00784-024-05542-4

**Published:** 2024-02-14

**Authors:** Pablo Machado Soares, Lucas Saldanha da Rosa, Rafaela Oliveira Pilecco, Amanda Maria de Oliveira Dal Piva, João Paulo Mendes Tribst, Arie Werner, Luiz Felipe Valandro, Gabriel Kalil Rocha Pereira, Cornelis Johannes Kleverlaan, Marilia Pivetta Rippe

**Affiliations:** 1 Post-Graduate Program in Oral Sciences, Center for Development of Advanced Materials, Division of Prosthodontics-Biomaterials, Federal University of Santa Maria (UFSM), Santa Maria, Brazil; 2 Department of Dental Materials Science, Academic Centre for Dentistry Amsterdam (ACTA), Universiteit Van Amsterdam and Vrije Universiteit, Amsterdam, North Holland the Netherlands; 3 Department of Reconstructive Oral Care, Academic Centre for Dentistry Amsterdam (ACTA), Universiteit Van Amsterdam and Vrije Universiteit, Amsterdam, North Holland the Netherlands

**Keywords:** Bond strength, Bulk-fill, Cyclic fatigue, Finite element analysis, Flowable resin, Resin composite

## Abstract

**Objective:**

The study aims to evaluate the shear bond and flexural strength fatigue behavior of yttrium-stabilized zirconia (4YSZ) repaired using different resin composites.

**Materials and methods:**

Cylindric specimens of 4YSZ were obtained for the bond strength (Ø = 6 mm, 1.5 mm of thickness) and biaxial flexural strength (Ø = 15 mm, 1 mm of thickness) fatigue tests and divided into 3 groups according to the repair resin composite: EVO (nanohybrid), BULK (bulk-fill), and FLOW (flowable). The zirconia surface was air-abraded with alumina particles, a 10-methacryloyloxydecyl dihydrogen phosphate (10-MDP) primer was applied, and the resin composite was build-up over the zirconia. Fatigue shear bond strength and flexural fatigue strength tests were performed (*n* = 15). One-way ANOVA and Tukey post hoc tests were carried out for both outcomes, besides scanning electron microscopy and finite element analysis.

**Results:**

The repair material affected the fatigue shear bond strength of zirconia ceramic. The BULK group (18.9 MPa) depicted higher bond strength values than FLOW (14.8 MPa) (*p* = 0.04), while EVO (18.0 MPa) showed similar results to both groups. No effect was observed for the mechanical behavior (*p* = 0.53). The stress distribution was similar for all groups.

**Conclusion:**

The repair of yttrium-stabilized zirconia (4YSZ) ceramics with bulk-fill resin composites was the best option for high fatigue bond strength. However, the fatigue mechanical performance was similar regardless of the applied repair material.

**Clinical relevance:**

The repair of yttrium-stabilized zirconia (4YSZ) monolithic restorations may be performed with nanohybrid and bulk-fill resin composites in order to promote longevity in the treatment.

## Introduction

Monolithic ceramic systems are currently considered one of the most effective restorative options for oral rehabilitation. Through the use of computer-aided design/computer-aided manufacturing (CAD/CAM), the production of such ceramic crowns is less time-consuming and decreases the presence of internal defects and technical variations when compared to the conventional bilayer systems [1–3]. Besides, previous studies reported higher mechanical performance and survival rates for monolithic restorations than for veneered zirconia [4, 5].

Among the dental ceramics systems indicated for monolithic restorations, yttrium-stabilized zirconia (4YSZ) stands out considering its mechanical performance and improved aesthetics provided by the advances of the third generation, with more translucent versions [6–8]. Soares et al. [7] reported that translucent zirconia restorations presented higher fatigue mechanical strength when compared to other ceramic materials such as lithium disilicate, zirconia-reinforced lithium silicate, and porcelain. These properties come from its polycrystalline arrangement, which combined with the increased cubic phase and different grain size provides a more translucent and high-performance material [8]. Even so, the brittle characteristic of dental ceramics and their susceptibility to crack initiation during fatigue stimulus can lead to the occurrence of technical complications such as chipping [9]; thus, clinicians and researchers are still looking for the best repair approach to overcome these failures [10].

Clinically it is essential to achieve both a stable bonding interface and a satisfactory mechanical behavior of the repaired restoration [11, 12]. In this sense, the interaction between zirconia and the repair resin composite must generate a well-filled and strongly bonded interface [13], while the microstructure of the repair material should also provide adequate load-bearing capacity during function. Several materials have been evaluated for direct repair applications, being the nanohybrid resin composite one of the most reported options [14, 15]. The resin matrix of this polymer combines nano- and micro-sized fillers, providing high mechanical strength, low polymerization shrinkage, and adequate polishing [14, 16].

Attempting to improve the results for repairing procedures, alternative resin-based materials have been suggested, as the use of bulk-fill and even flowable resin composites [17, 18]. The use of bulk-fill resin-based materials is increasing for restorative purposes as it makes the technique simpler by allowing the filling of larger cavities in a single increment (up to 4 mm) [19, 20]. It was reported that bulk-fill clinical stability was comparable to the use of conventional nanohybrid resin composites after 2 years [21]. Repair procedures with flowable resin composite have also been evaluated, due to its potential to fill superficial defects and provide tight contact with the restoration surface, showing promising results for bond strength with dental ceramics [22]. However, to the authors’ knowledge there is no current study comparing these restorative options when repairing a monolithic zirconia restoration, which is known as a material with low bonding potential when compared to the glass–ceramics [13]. Besides, the fatigue behavior for both bonding and mechanical performance of repaired translucent zirconia is still lacking in the literature.

Considering the aforementioned, the present study aimed to evaluate the fatigue shear bond strength and fatigue biaxial flexural strength of repaired 4YSZ when using three different resin composite materials (conventional nanohybrid, bulk-fill, and flowable resin composite) for the repair procedure. The assumed null hypotheses were that (1) the fatigue shear bond strength, (2) fatigue flexural strength of zirconia, and (3) the stress distribution would not be affected by the repair material.

## Materials and methods

The description of the utilized materials in the present study, including their commercial names, batch, and composition, are listed in Table 1.

**Table 1:** Description of the materials used in the study

Material	Commercial name	Manufacturer (batch number)	Main composition
Yttria-stabilized tetragonal zirconia polycrystal (4YSZ)	IPS e.max ZirCAD MT A2 shade	Ivoclar AG (V26180)	ZrO_2_; 8% weight of Y_2_O_3_; HfO_2_; Al_2_O_3_; other oxides
Aluminum oxide	White aluminum oxide 50 µm	Zest Dental Solutions (L2BN5)	50 µm Al_2_O_3_
MDP primer	Alloy primer	Kuraray Noritake Dental Inc. (210,121)	Acetone; 10-methacryloyloxydecyl dihydrogen phosphate (MDP); 6-(4-vinyl-benzyl-N-propyl) amino-1,3,5-triazine-2,4-dithione
Nanohybrid resin composite	Tetric EvoCeram A2	Ivoclar AG (Z03XMX)	Urethane dimethacrylate 5 < 10%; bis-GMA 3–7%; ytterbium trifluoride 3–5%; ethyoxylated bisphenol A dimethacrylate 3–5%
Bulk-fill resin composite	Tetric PowerFill A2	Ivoclar AG (Z03NPZ)	Bis-GMA, bis-EMA, UDMA, propoxylated bisphenol A dimethacrylate, DCP, β-allyl sulfone AFCT agent. Filler content: 77 wt%/54 vol%
Flowable resin composite	Tetric PowerFlow A2	Ivoclar AG (Z04DCX)	Bis-GMA, bis-EMA, UDMA. Filler content: 68 wt%/46 vol%

### Study design

Monotonic shear bond and biaxial strength pilot tests (*n* = 3) were performed in a universal testing machine (Instron 6022; Instron, Norwood, USA) at a crosshead speed of 0.5 mm/ min through a flat stainless-steel piston (Ø = 10 mm) for the bond strength test, and hemispheric piston (Ø = 1.6 mm) for the biaxial strength test, considering the factor under study (repair material) (95% confidence interval and statistical power of 80%). The OpenEpi statistical software program was used to calculate the sample size. Fifteen specimens were adopted for each fatigue test. The study design is described in Table 2.

**Table 2:** Experimental design

Group	Restorative set		Tests performed
	Zirconia	Repair material	
EVO	Translucent zirconia (4YSZ)	Repair with nanohybrid resin composite (Tetric EvoCeram, Ivoclar AG)	Fatigue shear bond strength test (*n* = 15) Fatigue biaxial flexural strength test (*n* = 15) Interface analysis (*n* = 1) Fractographic analysis (*n* = 1) Finite element analysis (*n* = 1)
BULK		Repair with bulk-fill resin composite (Tetric PowerFill, Ivoclar AG)	
FLOW		Repair with flowable resin composite (Tetric PowerFlow, Ivoclar AG)	

### Fatigue shear bond strength

#### Specimen’s preparation

Blocks of 4YSZ (IPS e.max ZirCAD MTA2, Ivoclar AG, Schaan, Liechtenstein) were obtained (10 × 10 × 16 mm) from a disk (blank) using a diamond disk coupled to a handpiece and an electric motor (Perfecta LA 623 T, 1000 to 40,000 rpm — W&H, Bürmoos, Austria). The blocks were attached to cylindrical metal guides and were grounded in a polishing machine (EcoMet/AutoMet 250, Buehler, Lake Bluff, USA) until reached a cylindrical form (Ø = 8 mm). Then, disks were obtained through slicing in a precision cutting machine (IsoMet 1000, Buehler) under water-cooling according to experimental tests, and were subsequently polished with silicon carbide (SiC) sandpapers #400-, #600-, and #1200-grit (3 M, Sumaré, Brazil). The specimens were sintered (Zyrcomat 6000 MS, VITA Zahnfabrik, Bad Sackingen, Germany) according to the manufacturer’s instructions (heating rate 10 °C/min until 900 °C; holding phase at 900 °C for 30 min; heating rate 3 °C/min until 1500 °C; holding phase at 1500 °C for 120 min; cooling rate 8 °C/min), assuming then their final dimensions (Ø = 6 mm, 1.5 mm of thickness).

#### Surface treatment and bonding procedures

As a first step, a grinding protocol with diamond bur was performed (4219F, 46-µm grain size, KG Sorensen, Cotia, São Paulo, Brazil) to simulate a clinical scenario, where the surface is roughened after fracturing and before the repair with direct resin composite. A permanent mark was made on the specimen’s surface to standardize the grinding process. The bur was coupled to a multiplier contra-angle (T2 REVO R170 contra-angle handpiece up to 170,000 rpm, Sirona, Bensheim, Germany), and the grinding was performed (3 specimens per diamond bur) with oscillatory movements underwater cooling and parallel to the surface of the specimens until the pen mark was completely removed [23].

After that, the specimens were cleaned in an ultrasonic bath for 5 min, and air-abraded with alumina particles at 10-mm distance, with 2.8 bar of pressure for 10 s [24]. A 10-MDP primer (Alloy Primer, Kuraray Noritake Dental Inc.) was applied actively over the surface of the specimens for 10 s and gently air-dried, according to the manufacturer’s recommendations.

The specimens were fixed in a metallic device for the fatigue shear bond strength test. To reduce the bonding area between zirconia and the repair resin-based material, adhesive tapes (Scotch Magic Tape, 3 M, Saint Paul, USA) were positioned 1 mm apart over the zirconia disk, being this distance controlled with a digital caliper (Absolute digimatic, Mitutoyo, Kawasaki, Japan) [25]. The second metallic pair device was standardly positioned against the first one by using a polyvinyl siloxane matrix (Express XT Putty, 3 M ESPE, Seefeld, Germany), and then the repair resin composite material was applied over the zirconia specimens according to the factor under study, as described in Table 2: nanohybrid resin composite, bulk-fill resin composite, and flowable resin. The resin-based materials were applied in one increment (Ø = 6 mm, 1.5 mm of thickness; interface: 1 mm) and then light cured at 1200 mW/cm^2^ (Radii-cal LED curing light, SDI, Bayswater, Australia) for 20 s. The specimens were stored at 37 °C for at least 24 h until the fatigue shear bond strength test.

#### Fatigue shear bond strength test

A fatigue shear bond test (*n* = 15) was performed in an adapted equipment (ACTA, Amsterdam, Netherlands) that uses a pneumatic system [25]. The metallic apparatus was vertically positioned in a base, with the load being applied through a flat stainless-steel load piston (Ø = 13 mm) over the device, which consequently acted on the bonding interface of the specimen. A 200-N load cell was used for the test. The fatigue test parameters were initial load of 10 N during 10,000 cycles, followed by steps of 10 N/10,000 cycles each, with frequency of 2–3 Hz until the failure occurred. The fatigue failure load (FFL) and cycles for failure (CFF) data were recorded and the fatigue shear bond strength “*FS*” (in MPa) was calculated (*FS* = *L*/*A*), being “*L*” fatigue failure load (in Newtons) and “*A*” the cross-sectional area of the interface that was calculated for each specimen considering the bonding area (overall mean = 5.8 mm^2^).

#### Failure analysis

The failed specimens were analyzed under a stereomicroscope (Discovery V20, Carl Zeiss, Gottingen, Germany) with a magnification of 15 × . The failures at the zirconia/resin composite bonding interface were categorized as predominantly adhesive (˃50% of the failures were adhesive through the adhesive interface) or predominantly cohesive (˃50% were cohesive within the ceramic or the resin composite material) [25]. One representative specimen of each group was selected for a scanning electron microscopy (SEM; Evo LS15, Carl Zeiss, Gottingen, Germany) evaluation with magnification of 100 × .

### Flexural fatigue strength

#### Specimen’s preparation

Similar procedures that those aforementioned for the shear test were adopted to obtain the 4YSZ disks (*n* = 15; Ø = 15 mm, 1-mm thickness). All specimens were also grounded with diamond burs, air abraded with alumina particles [23, 24], treated with a 10-MDP primer (Alloy Primer, Kuraray Noritake Dental Inc.) for 10 s, and then gently air-dried.

The resin composites were applied over the treated zirconia disks using a polyvinyl siloxane matrix (0.4 mm of thickness). A glass plate was pressed against the resin-based material, and the light curing was performed at 1200 mW/cm^2^ (Radii-cal LED curing light, SDI, Bayswater, Australia) for 20 s. The top surface of the repair resin composite was polished with SiC sandpaper #600- and # 1200-grit until the standard final thickness (0.4 mm for the repair, 1 mm for zirconia; total thickness = 1.40 mm ± 0.02 mm). The thickness was double-checked with a digital caliper (Absolute digimatic, Mitutoyo, Kawasaki, Japan), being replaced any specimen that did not reach the desired thickness. The specimens were stored in distilled water for at least 24 h at 37 °C before the biaxial fatigue test.

#### Biaxial flexural fatigue strength test

A cyclic fatigue biaxial test was performed in a fatigue testing machine (ACTA, Amsterdam, Netherlands), through a steel piston (Ø = 1.6 mm) positioned at the center of the top surface of each specimen, with the zirconia layer facing down. The specimens were positioned on a base with 3 equidistant spheres (10 mm) and tested in distilled water [26]. An adhesive tape was positioned on the top surface of the repair resin composite to promote a more homogenous stress distribution and to keep the fragments together after fracture. The tested parameters were initial load of 100 N for 10,000 cycles, step size of 25 N, and 10,000 cycles/step using 1.4 Hz of frequency until the fracture occurs. After each test, the FFL and CFF data were obtained and used for statistical purposes.

#### Failure and interface analysis

The fractographic analysis was performed under a stereomicroscope (Discovery V20, Göttingen, Carl Zeiss), and one representative specimen from each group was selected for a SEM analysis at 150 × and 500 × magnifications, to evaluate the effect of the repair material both in the bonding interface aspect and for the failure origin characterization.

### Finite element analysis

**Fig. 1:**
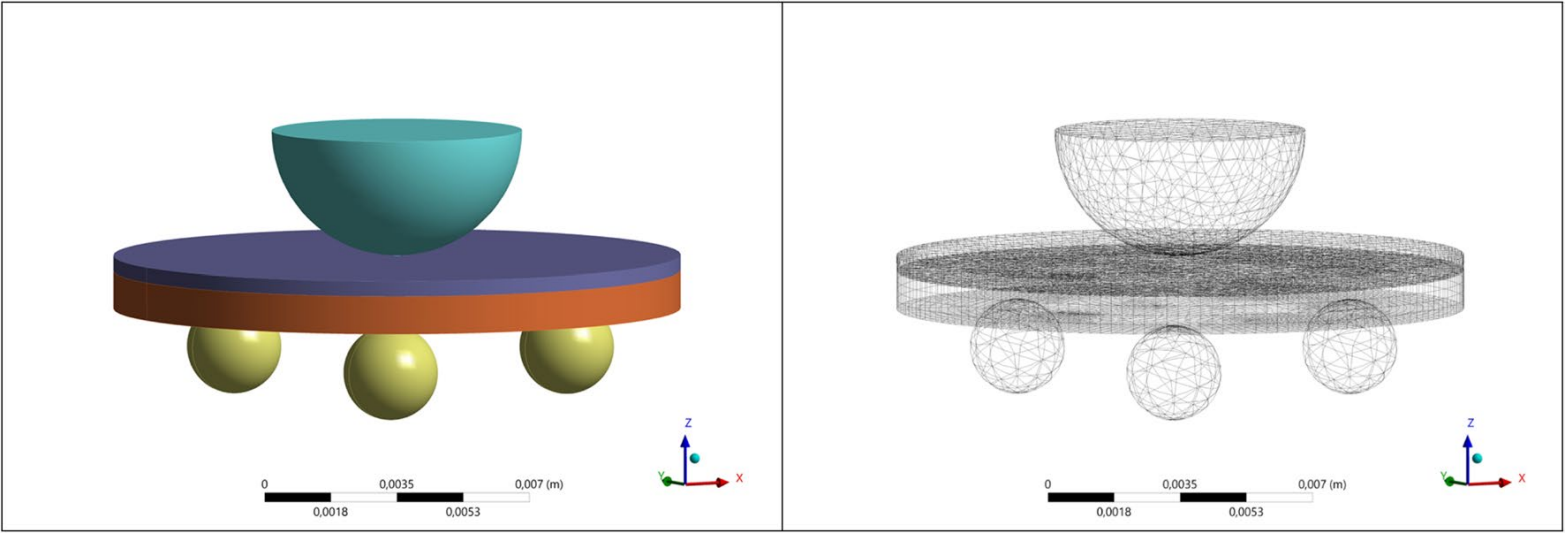
Specimen model for the finite element analysis (left) and mesh (right). The orange region indicates the zirconia material, which was at the bottom during the fatigue test and positioned over the metal spheres (yellow balls). The purple zone of the specimen is the resin composite repair, which was at the top and in contact with the load applicator (blue hemisphere)

The stress distribution and fatigue strength (in MPa) on the center of the repaired ceramic during the mechanical test were evaluated by a three-dimensional (3D) finite element analysis (FEA). For that, three models (Fig. 1) of the repaired zirconia disks presenting the same dimensions of the in vitro test (Ø = 15 mm, 1.4-mm thickness) were digitally obtained (Rhinoceros, version 5.0 SR8, McNeel North America), considering the flexural strength test (specimen, base, and load applicator). The evaluated materials’ elastic modulus (*E*) and Poisson ratio (*v*) were used for the analysis (zirconia − *E* = 200 GPa, *v* = 0.31 nanohybrid resin composite − *E* = 11 GPa, *v* = 0.3; bulk-fill resin composite − *E* = 8.4 GPa, *v* = 0.30; flowable resin − *E* = 5.6 GPa, *v* = 0.30; stainless-steel ring/sphere − *E* = 190 GPa, *v* = 0.27), according to manufacturers and previous studies [7, 27]. The materials were considered isotropic, linear, and homogeneous. Boundary conditions were applied to simulate in-vitro constraints on the disk structure. The elemental constraints were considered to replicate support locations. These constraints restricted the displacement and rotation of selected nodes in all directions. The elemental loading (200 N) comprised tailored force distributions applied directly to individual elements on the surface of the loading sphere and was used for the mesh convergence test. Then, the fatigue failure load (FFL) data obtained in-vitro was used to define the fatigue strength of each group considering the stress calculated by FEA.

In the simulated linear scenario, no attrition was considered between the supporting rods, loading applicator, and the testing specimens. A 10% mesh convergence test was assumed in the results to evaluate the differences in strength between the groups [28]. The data analysis was performed by the use of a computer-aided engineering software program (ANSYS 19, ANSYS Inc., Houston, USA), to determine the maximal principal stress (MPa).

### Statistical analysis

Normality and homoscedasticity evaluations were performed both for shear and biaxial fatigue data through Shapiro–Wilk and Levene tests, respectively (*p* > 0.05). One-way analysis of variance (ANOVA) and Tukey post hoc tests (*α* = 0.05) were performed to evaluate the effect of the repair material factor on the fatigue performance, using the SPSS statistical program (21 version, IBM, Chicago, USA). Kaplan–Meier and Mantel-Cox post hoc (log-rank) tests were also performed for the determination of the survival rates (*α* = 0.05) of the fatigue shear and biaxial data.

## Results

**Fig. 2:**
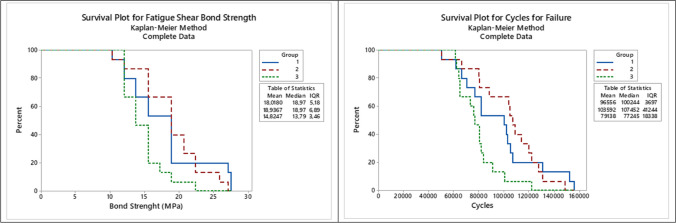
Survival plots depicting the fatigue behavior for fatigue shear bond strength test (Group 1: EVO; Group 2: BULK; Group 3: FLOW), considering the shear bond strength and number of cycles

**Fig. 3:**
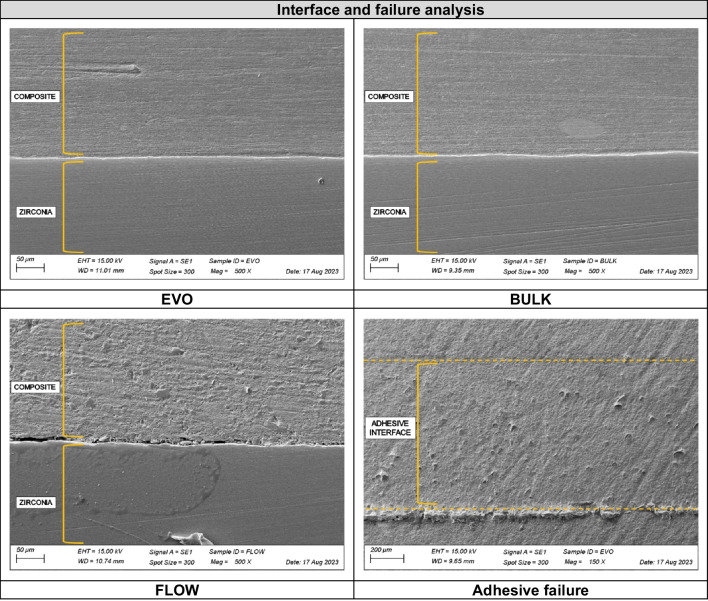
SEM images of the interface analysis according to the repair material. Representative SEM image of the adhesive failures depicted by the fatigue shear bond strength test

The results of the fatigue shear bond strength (*FS*) test are depicted in Table 3. One-way ANOVA showed that the resin composite material affected the shear bond strength of repaired zirconia (*FS*: *p* = 0.04, *F* = 3.36; CFF: *p* = 0.03, *F* = 3.66), with the BULK group presenting the highest values of bonding, while FLOW group having the lowest adhesion values. When repaired with nanohybrid resin composite, the fatigue bond strength was similar to both BULK and FLOW groups. These findings are corroborated by the survival rates (Fig. 2), since after 15 MPa of inducted stress, 63% of the BULK specimens and 50% of the EVO group survived, while only about 20% of survival was observed for the FLOW group. Besides, all failures were considered predominantly adhesives (Fig. 3), as represented by the SEM images.

**Table 3:** Mean (95% confidence interval) results for the fatigue shear bond and flexural strength tests according to each group

Groups	Fatigue shear bond strength test		Biaxial flexural fatigue strength test		
	Bond strength (MPa)	Cycles for failure (CFF)	Load for failure (FFL)	Cycles for failure (CFF)	Fatigue strength (MPa)^*^
EVO	18.0 (15.2–20.9)^AB^	96,555 (80,628–112,482)^AB^	343.33 (310.39–376.28)^A^	81,131 (67,687–94,574)^A^	458^A^
BULK	18.9 (16.6–21.3)^A^	103,591 (90,295–116,887)^A^	368.33 (342.82–393.85)^A^	89.368 (78,882–99,853)^A^	466^A^
FLOW	14.8 (13.3–16.3)^B^	79,138 (70,841–87,434)^B^	348.33 (310.84–385.82)^A^	82,987 (67,505–98,468)^A^	475^A^

Mean (95% confidence interval) fatigue failure load, cycles for failure, and flexural strength (MPa) result for the fatigue biaxial flexural strength test. Capital letters show the significant differences between the groups for each analysis (columns) depicted by Kaplan–Meier and log-rank (shear bond strength and flexural fatigue tests) tests

^*^The differences in fatigue strength between the groups were considered significant when higher than 10% considering the mesh convergence

**Fig. 4:**
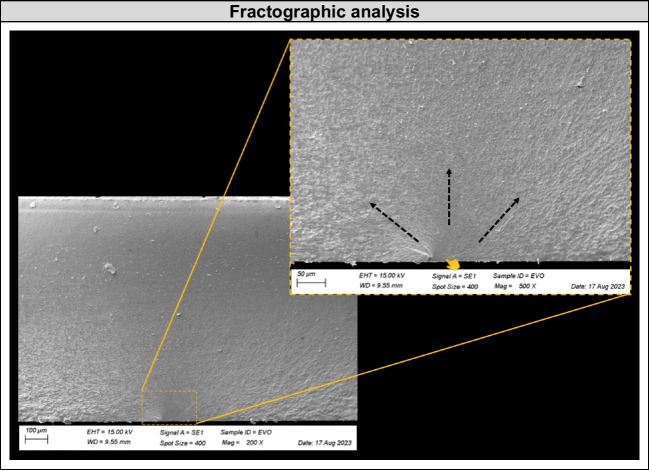
Fractography analysis of a representative specimen. All failures originated in the zirconia bottom surface during the biaxial flexural fatigue strength test

The biaxial flexural fatigue strength test results are depicted in Table 3 and 4. No influence of the tested repair material was noticed by one-way ANOVA (FFL: *p* = 0.53, *F* = 0.64; CFF: *p* = 0.62, *F* = 0.49). Thus, all evaluated resin composites presented similar mechanical fatigue performance when repairing zirconia ceramics. SEM revealed that the interface analysis showed a higher presence of flaws and defects between zirconia and flowable resin, while the interface was more well-filled and homogenous when nanohybrid and bulk-fill resin composites were used for the ceramic repair (Fig. 3). The fractography analysis showed that the crack initiated at the bottom of the zirconia, which was under tensile stress during the cyclic fatigue test (Fig. 4).

**Table 4:** Percentage of survival rates (standard error) indicating the probability of the specimens exceeding the respective fatigue failure load (FFL) and cycles for failure (CFF) step without failure during the mechanical test, according to each repair material

Groups	FFL (N)/CFF										
	175/20,000	200/30,000	225/40,000	250/50,000	275/60,000	300/70,000	325/80,000	350/90,000	375/100,000	400/110,000	425/120,000
EVO	1	1	1	0.93 (0.06)	0.80 (0.10)	0.53 (0.13)	0.40 (0.13)	0.40 (0.13)	0.40 (0.13)	0.27 (0.11)	0
BULK	1	1	1	0.93 (0.06)	0.93 (0.06)	0.87 (0.09)	0.80 (0.10)	0.60 (0.13)	0.33 (0.12)	0.27 (0.11)	0
FLOW	1	0.93 (0.06)	0.93 (0.06)	0.80 (0.10)	0.73 (0.11)	0.67 (0.12)	0.67 (0.12)	0.60 (0.13)	0.40 (0.13)	0.20 (0.10)	0

**Fig. 5:**
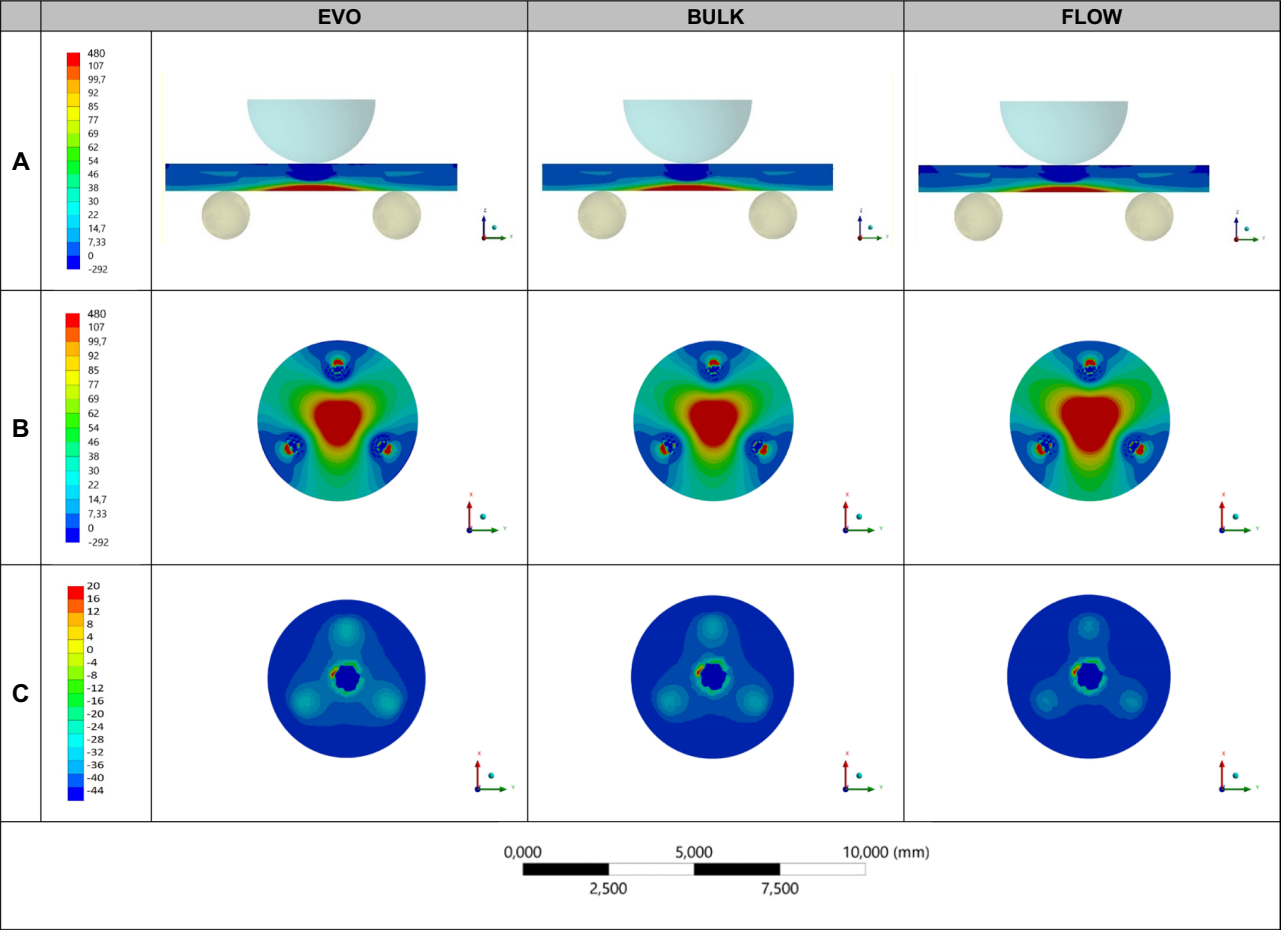
Finite element analysis (**A** section planes with the zirconia facing down, **B** zirconia material, and **C** repair material at the top) of the repaired zirconia specimens according to each group: EVO (nanohybrid), BULK (bulk-fill), and FLOW (flowable)

**Fig. 6:**
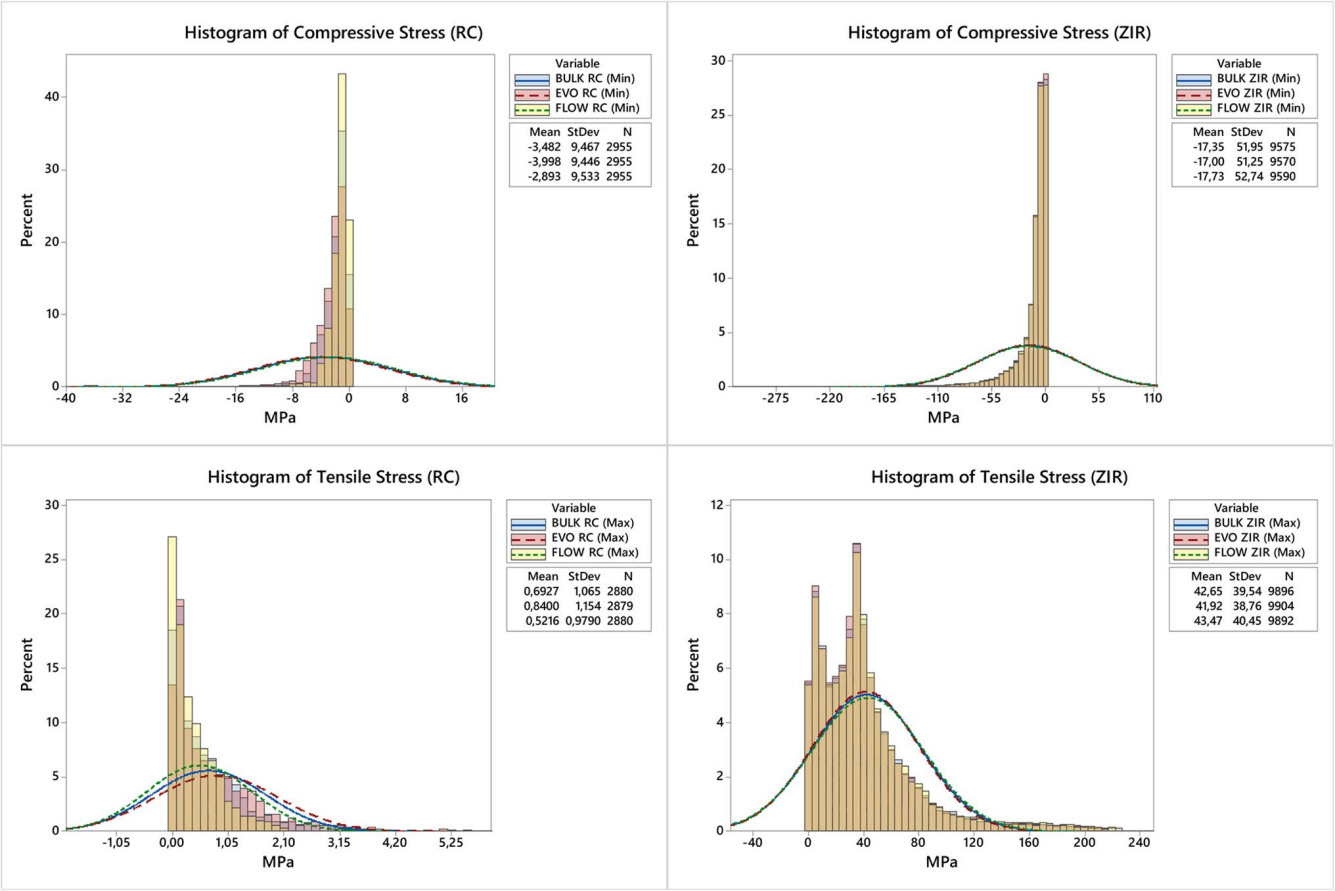
Histogram from element stress according to maximum principal stress (tensile) and minimal principal stress (compression) of the repair material (RC — left) and repaired zirconia (ZIR — right) for each restorative material: EVO (nanohybrid), BULK (bulk-fill), and FLOW (flowable)

The finite element analysis is depicted in Fig. 5. The maximal principal stress (MPa) was similar for all models, regardless of the repair material used. Similar patterns of stress distribution were observed in the zirconia ceramic, while less compressive stress concentration was present in the flowable resin when compared to the other repair materials. The stress distribution across the surface has been obtained through surface graphs, with stress per element presented in histograms for both compression and tensile stresses across each simulated condition (Fig. 6). In summary, the histogram of stress per element represents the distribution of stresses experienced by individual elements within the analyzed structure (substrate or repair). If the histogram bars are relatively uniform in height across various stress levels, it might suggest a more even distribution of stresses among the elements, indicating a more balanced stress pattern within the structure. On the other hand, a longer data spread along the *x*-axis in the histogram suggests a wider range of stress magnitudes experienced by different elements.

## Discussion

In this study, the fatigue bond strength between zirconia ceramic and the repair material was affected by the used resin composite, since the bulk-fill composite presented higher values of bonding when compared to the flowable resin, while the nanohybrid material presented similar results to both BULK and FLOW groups. Thus, the first null hypothesis was rejected. These findings may be explained by the filler content in the microstructure of the bulk-fill resin composite, which presents photoactive groups disposed within methacrylate, and, thus, improved polymerization kinetics, allowing the use of bigger increments up to 4 mm [19]. This is in accordance with a previous study that compared bulk-fill and methacrylate-based flowable composites, showing that the bulk-fill presented a higher degree of polymerization and bonding capacity than flowable composites [20].

The lower filler content of flowable resin was previously associated with the lower potential of bonding and greater polymerization shrinkage when compared to conventional resin composites [20, 29–31]. SEM images of the interface analysis corroborate these findings, where the repaired interface of the FLOW group presents more gaps and defects when compared to the conventional nanohybrid and bulk-fill resin composites (Fig. 3). The nanohybrid repair material also presented high values of fatigue bond strength; however, they were similar to both bulk-fill and flowable resin. As well as bulk-fill composites, conventional nanohybrid materials also contain more filler particles than flowable resin [29]; however, its lower degree of polymerization probably explains the intermediate values of bond strength during the fatigue test. Besides, Dačić et al. reported no difference in bond strength when comparing conventional and bulk-fill groups, thus corroborating the findings of the current study [32].

Regarding the mechanical behavior of the repaired zirconia in fatigue, no difference was observed between the evaluated groups according to the repair resin composite (Table 3). Thus, the second null hypothesis was accepted. Despite the different filler content and bonding potential of each repair resin-based material [20, 29, 30], it seems that these differences were not enough in terms of the mechanical behavior of the entire restorative set to generate distinct reinforcement effects during the flexural fatigue test.

A previous study showed that the material under tensile stress (facing down during the flexural test) dictates the mechanical behavior and also the failure mode of multilayered sets [33]. Indeed, in our study the repaired zirconia specimens presented similar patterns of failure for all groups, being the fracture origin located at the bottom surface of the zirconia (Fig. 4), which was under more stress during cyclic load application. After the first crack, the failure propagated towards the repair resin composite at the top surface until the complete fracture of the specimen. These findings are corroborated by the finite element analysis (Fig. 5), which illustrated such higher tensile stress concentration at the bottom and middle of the ceramic, regardless of the repair material. Besides, the third hypothesis was also accepted since similar stress values were observed for all groups (Table 3), even with a slight difference in stress distribution at the top surface of the repair material, with lower compressive stress for the flowable resin, as expected due to the lower elastic modulus of this material. These analyses may explain the similar fatigue strength found for all groups, which were tested with the repair material at the top and under compression during the mechanical test to simulate a clinical scenario.

Enhancing the bonding potential of zirconia is a constant challenge due to its polycrystalline microstructure and the almost total absence of silica content [8]. In this sense, the statement of a standard protocol of repair for this material is still lacking, and different resin-based materials have been suggested to repair zirconia crowns with a high and stable level of bonding, thus assuring longevity for repaired restorations [17, 18]. The present study adopted the use of a 10-MDP containing primer, which was previously reported as an effective option due to the action of the phosphate monomer to increase the bonding potential of zirconia by a chemical mechanism, mainly when combined with mechanical interlocking promoted by air abrasion [34, 35]. A previous study showed that a primer with at least 0.5 wt % of each monomer in the composition significantly improved zirconia bonding (Alloy primer) [35]. This balanced combination compensates for zirconia’s lack of a glass phase and chemical inertness, ensuring durable bonding with resin materials [35]. To evaluate the bond strength stability, this study adopted a shear bond strength test in a cyclic fatigue regime, which was previously described in a previous study [25]. The used approach was effective in evaluating the cumulative effect of cyclic loads on the bonded interface between lithium disilicate and dentin substrates, thus evaluating the bonding stability of repaired zirconia ceramics [36], which is closer to the clinical scenario. This methodology was also considered effective in the present study for the bonding outcome since all the failures were classified as predominantly adhesive (Fig. 3).

In a clinical scenario, it must also be considered the suitability of the application for each repair resin composite. The handling of conventional and bulk-fill materials is quite similar; however, the advantage of applying increments up to 4 mm makes bulk materials more feasible for the repair of posterior crowns when the access of the failure is more difficult or deeper [19, 20]. On the other hand, the use of flowable resin composite may be more difficult depending on the type of repair, due to its low viscosity depicted by the lower filler content when compared to the conventional resin composites [29]. Thus, for instance, the repair of cusp failures with a flowable material could be challenging.

As limitations of the present study, it must be highlighted that the specific stress value for each tested specimen was not measured, due to the programming constraint during the mechanical tests. Thus, the fatigue failure load data were obtained, and used by the FEA to define the fatigue strength of each group (Table 3). Another limitation is the lack of thermocycling after the specimen preparation. Thermocycling is an important method to evaluate the bonding stability and interface integrity of dental materials after aging. However, the authors believe that the performed fatigue shear bond strength and fatigue flexural strength tests were effective methods to evaluate the bonding and mechanical behavior of repaired zirconia in terms of longevity and survival, since the main reason of failure for restorations in the clinical scenario is the fatigue stimulus. Furthermore, the reproducibility of the fatigue bond strength method is difficult since it relies on the use of very specific equipment, which is not available in the market. However, the test was essential to show the cumulative effect of cyclic loads at the bonding interface, which is closer to the evaluation of restoration longevity in a clinical scenario. Finally, the repaired zirconia was not tested on a specific substrate, which may affect the mechanical performance of the restoration when luted. Thus, future studies should simulate the fatigue behavior of tooth and implant-supported restorations, to consider the influence of different substrates on the repaired zirconia restoration performance.

## Conclusion

Since the mechanical performance of repaired translucent zirconia was not affected by the used resin composite, the repair material should be selected based on its adhesive properties. In the simulated conditions, due to the high bond strength performance in fatigue, both conventional nanohybrid and bulk-fill resin composites may be alternatives for the repair of monolithic translucent zirconia.
